# Therapeutic Potential of Saikosaponins in MASLD and Cross‐Organ Protection: A Research Update

**DOI:** 10.1002/fsn3.71470

**Published:** 2026-02-08

**Authors:** Jiayao Xiong, Wanyan Tan, Ze Jin, Xiangyu Li, Yuting Chen, Wanting Xie

**Affiliations:** ^1^ Department of Gastroenterology, Liyuan Hospital, Tongji Medical College Huazhong University of Science and Technology Wuhan China

**Keywords:** cross‐organ protection, mechanism, metabolic dysfunction‐associated steatotic liver disease, saikosaponins, traditional Chinese medicine

## Abstract

Metabolic dysfunction‐associated steatotic liver disease (MASLD), formerly known as non‐alcoholic fatty liver disease (NAFLD), has emerged as the most prevalent chronic liver disorder in China, posing a significant threat to public health. While current Western pharmacological therapies demonstrate efficacy, they don't comprehensively ameliorate pathological components of MASLD and are often associated with substantial adverse effects. Saikosaponins (SSs), as the main active components of *Bupleurum chinense*, a traditional Chinese medicine (TCM), have been demonstrated to exert potential therapeutic effects on MASLD in recent years. However, there is a lack of systematic integration of their mechanisms of action and clinical evidence. This review aims to summarize the molecular mechanisms, clinical research progress, and existing challenges regarding the regulation of MASLD by SSs and systematically reviews the multiple therapeutic mechanisms of SSs, including the regulation of lipid metabolism, improvement of insulin resistance, exertion of anti‐inflammatory and antioxidant effects, modulation of intestinal flora, and inhibition of fibrosis, while summarizing the clinical applications of its compound formulas. Additionally, this article summarizes the cross‐organ protective effects of SSs in the liver, cardiovascular system, kidneys, immune system, and central nervous system, providing further theoretical references for the treatment of MASLD and its complications.

## Graphical abstract

This review systematically outlines the multi‐target mechanisms of Saikosaponins (SSs) against MASLD, encompassing the regulation of lipid metabolism, insulin resistance, and inflammatory response. Furthermore, it highlights their novel cross‐organ protective effects on the cardiovascular, renal, and central nervous systems, providing therapeutic strategies for MASLD and its systemic complications.

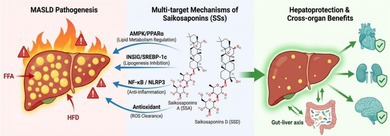

## 1 Introduction

MASLD is a hepatic manifestation of metabolic syndrome, characterized by liver fat accumulation in the presence of one or more cardiometabolic risk factors and the absence of significant alcohol consumption (Tacke et al. 2024). In 2023, a global panel of fatty liver experts reached a consensus to rename NAFLD as MASLD. Unlike the exclusion‐based diagnosis of NAFLD, MASLD establishes clear diagnostic criteria requiring: (1) hepatic steatosis affecting > 5% of hepatocytes, (2) presence of at least one of the following five core cardiometabolic risk factors:

1. Body mass index (BMI) ≥ 25 kg/m^2^ (≥ 23 kg/m^2^ for Asian populations) or waist circumference (> 94 cm for men, > 80 cm for women), with adjustments for race/ethnicity.
2. Fasting plasma glucose ≥ 5.6 mmol/L (≥ 100 mg/dL) or 2‐h postprandial glucose ≥ 7.8 mmol/L (≥ 140 mg/dL) or HbA1c ≥ 5.7% or diagnosed type 2 diabetes mellitus.
3. Blood pressure ≥ 130/85 mmHg or current use of antihypertensive medications.
4. Plasma triglycerides ≥ 1.70 mmol/L (150 mg/dL) or current lipid‐lowering therapy.
5. Plasma HDL‐cholesterol ≤ 1.0 mmol/L (men) or ≤ 1.3 mmol/L (women) or current lipid‐lowering therapy (Rinella et al. 2023).

In fact, an international panel of fatty liver experts had proposed renaming NAFLD to MAFLD in 2020 (Eslam, Newsome, et al. 2020; Eslam, Sarin, et al. 2020). Song et al.'s population‐based study demonstrated that research findings related to NAFLD remain valid under MASLD criteria, with minimal differences in metabolic parameters between the two classifications (Hagström et al. 2024; Song et al. 2024). A 2023 meta‐analysis revealed that the global prevalence of MASLD increased from 25.3% (1990–2006) to 30% in 2019, showing a continuous upward trend. Over the past three decades, the global prevalence has risen by nearly 50% (Younossi et al. 2023). The progression of MASLD follows a sequential pathogenesis: it initially manifests as simple steatosis, which—if left untreated—can advance to metabolic dysfunction‐associated steatohepatitis (MASH), subsequently progressing to liver fibrosis and cirrhosis, ultimately potentially culminating in hepatocellular carcinoma. Throughout this continuum, the degree of hepatic inflammation, hepatocellular injury, and fibrosis progressively worsens (Eslam, Newsome, et al. 2020; Kaya and Yilmaz 2022; Tacke et al. 2024). Notably, MASLD, as a hepatic manifestation of metabolic syndrome, is closely associated with various extrahepatic diseases, including obesity, cardiovascular disease (CVD), type 2 diabetes mellitus (T2DM), chronic kidney disease (CKD), hyperlipidemia, systemic adipose tissue dysfunction and inflammation, and extrahepatic malignancies. Particularly, the 2023 American Heart Association (AHA) concept of “cardiovascular‐kidney‐metabolic syndrome (CKM)” further highlights the significance of MASLD in systemic metabolic disorders and the importance of extrahepatic organ protection. Early preventive measures and screening can reduce complications and lower mortality rates (Karlsen et al. 2022; Khan et al. 2023).

This review employed the following literature search and selection methodology: Relevant studies were systematically searched in PubMed, Embase, Web of Science, CNKI, Wanfang Data, and VIP databases, encompassing literature published within the past 15 years, with 70% of the included studies published in the last 5 years. The search keywords included “Saikosaponins,” “*Bupleurum chinense*,” “MASLD,” “NAFLD,” “NASH,” “MASH,” “liver fibrosis,” “mechanism,” “clinical trial,” “Cardiovascular complications,” “Extrahepatic cancers,” “hepatocellular carcinoma,” “chronic kidney disease,” “obesity,” “type 2 diabetes mellitus,” “hyperlipidemia,” “immune system,” and “central nervous system.” The inclusion criteria were defined as: (1) studies focusing on the mechanisms of action, pharmacological effects, or clinical applications of saikosaponins in MASLD; (2) original research (including basic experiments, animal studies, and clinical trials) or high‐quality systematic reviews/meta‐analyses; (3) articles published in Chinese or English with full‐text availability; and (4) published experimental patents. Exclusion criteria included: (1) abstracts, conference proceedings, or literature with incomplete data; (2) studies unrelated to saikosaponins or MASLD; and (3) literature with obvious methodological flaws or insufficient scientific rigor. Two researchers independently screened the literature by title, abstract, and full text, with any discrepancies resolved through consultation with a third researcher. The quality of evidence for the included studies was evaluated using the criteria of the Oxford Centre for Evidence‐Based Medicine.

Currently, lifestyle modifications is mainly weight loss, dietary changes, physical exercise, alcohol cessation, and bariatric surgery to MASLD. A rigorously controlled intervention trial has shown that a weight reduction of ≥ 5% can decrease hepatic lipid content, 7%–10% can improve inflammation, and ≥ 10% can ameliorate liver fibrosis. However, these interventions are often unable to do it due to poor execution, low adherence, and insufficient clinical data (Wei et al. 2024). According to the recommendations in the latest guidelines, the core therapeutic drugs used in clinical practice for MASLD include: obeticholic acid and remitirom; pioglitazone, Glucagon‐like Peptide‐1 Receptor Agonist (GLP‐1 RA), and Sodium‐Glucose Cotransporter 2 Inhibitor (SGLT2i); statins and vitamin E (Tacke et al. 2024). Obeticholic acid is the first drug proven effective for non‐cirrhotic MASH‐related liver fibrosis in phase III trials, with a clear effect on reversing liver fibrosis. However, attention should be paid to its side effects such as pruritus, lipid disorders, and cardiovascular diseases. Remitirom has been approved for marketing by the Food and Drug Administration (FDA) in March 2024, but it is only approved for patients with F2–F3 stage liver fibrosis (Ledford 2024). Its applicability to MASLD patients at other stages remains to be verified, and due to its short time on the market, the authenticity of relevant data is limited. GLP‐1 RA, SGLT2i, pioglitazone, and statins are mainly used in patients with “MASLD complicated with type 2 diabetes/obesity/hyperlipidemia.” They indirectly protect the liver by improving the underlying diseases and simultaneously reduce the risk of extrahepatic (cardiovascular and renal) outcomes. However, they are only recommended by guidelines, may cause various side effects, and have limited therapeutic effects. Vitamin E is only applicable to the specific subgroup of patients with “no diabetes and persistently elevated Alanine Aminotransferase (ALT)” and is not recommended as a routine medication. MASLD is a complex metabolic disease, and there is currently no single drug that can comprehensively improve all its pathological components (Sangro et al. 2023). Meanwhile, with the recent resurgence of interest in TCM, the integrated therapeutic effects of TCM monomers and compound prescriptions have demonstrated remarkable advantages for MASLD treatment. These preparations exhibit synergistic actions through multi‐target and multi‐pathway mechanisms, which align well with the complex pathogenesis of the disease (Dai et al. 2021; Shi et al. 2020).

SSs primarily extracted from plants of the Bupleurum genus, are triterpenoid saponins with a pentacyclic oleanane‐type skeleton. To date, over 100 saponin components have been isolated, with the major constituents being saikosaponin A (SSA), saikosaponin B1 (SSB1), saikosaponin B2 (SSB2), saikosaponin B3 (SSB3), saikosaponin B4 (SSB4), saikosaponin D (SSD), and saikosaponin C (SSC). These compounds exhibit distinct molecular structures but demonstrate diverse biological activities, including anti‐inflammatory, hepatoprotective, antioxidant, immunomodulatory, and antitumor effects. The shared triterpenoid skeleton among these compounds results in similar biological activities across different molecules. Notably, SSA and SSD exhibit the strongest biological activities, manifesting the most pronounced pharmacological effects among all SSs (Biswas and Dwivedi 2019; He et al. 2019; Li et al. 2018). According to the Chinese Pharmacopeia (2020 Edition), the primary active components in Bupleurum are SSA and SSD, with the combined content of these two components required to be no less than 0.30% (National Pharmacopoeia Commission 2020). In terms of anti‐inflammatory effects, both SSA and SSD demonstrate significant anti‐inflammatory properties, primarily by inhibiting the NF‐κB and MAPK signaling pathways, thereby reducing the production of pro‐inflammatory cytokines including nitric oxide (NO), prostaglandin E2 (PGE2), and tumor necrosis factor‐alpha (TNF‐α) (Lu et al. 2012; Yuan et al. 2017). In terms of anti‐tumor effects, SSD exhibits stronger anti‐cancer properties primarily through two mechanisms: (1) inhibiting cyclooxygenase‐2 (COX‐2) expression and activating caspase‐dependent apoptosis, and (2) modulating the p38 signaling pathway to exert its anti‐proliferative effects on tumor cells (Cai et al. 2017; Maik‐Rachline et al. 2020; Martínez‐Limón et al. 2020). Furthermore, studies have demonstrated that SSD shares structural similarity with estrogen and exerts additional anti‐tumor effects by binding to estrogen receptor alpha, thereby mimicking estrogen's physiological actions. This estrogen receptor‐mediated mechanism contributes to its overall anti‐cancer properties (Wang et al. 2010).

## 2 Mechanisms of SSs in Treating MASLD

### 2.1 Regulation of Lipid Metabolism

The pathogenesis of MASLD typically begins with dysregulation of lipogenesis or fatty acid oxidation, leading to excessive lipid accumulation in hepatocytes. Saikosaponins can ameliorate the fatty acid metabolic disorder in MASLD through multiple molecular targets. Adenosine monophosphate‐activated protein kinase (AMPK), which plays a crucial role in energy homeostasis regulation, is particularly important in metabolically active tissues such as the liver, adipose tissue, and skeletal muscle (Ha et al. 2011). In Gu et al.'s study, intragastric administration of SSA to high‐fat diet‐induced MASLD rats for 14 consecutive days significantly decreased fasting blood glucose levels (*p* < 0.05) and hepatic triglyceride content (*p* < 0.01). Mechanistic analysis revealed that SSA exerts its lipid‐lowering effects by activating the AMPK/PPARα signaling pathway, thereby improving hepatic lipid metabolism (Gu et al. 2021). Gu et al.'s study demonstrated that SSD upregulates the expression of insulin‐induced genes (INSIG1/2), thereby inhibiting the translocation of sterol regulatory element‐binding protein 1c (SREBP1c) from the endoplasmic reticulum (ER) to the Golgi apparatus and downregulating the expression of its downstream target genes, including fatty acid synthase (FASN) and acetyl‐CoA carboxylase (ACACA). This molecular cascade suppresses de novo lipogenesis, reducing hepatic triglyceride (TG) accumulation. Experimental evidence further confirmed that both SSA and SSD exerted significant, dose‐dependent effects in lowering serum TG, total cholesterol (TC), and hepatic enzyme levels (alanine aminotransferase, ALT and aspartate aminotransferase, AST), while attenuating intrahepatic lipid droplet deposition. Notably, these metabolic improvements occurred without altering food intake, indicating direct pharmacological regulation of lipid metabolism. Importantly, SSD displayed superior hypolipidemic efficacy compared to SSA in these preclinical models (Alves‐Bezerra and Cohen 2017; Gu et al. 2022). SSD acting as a natural agonist of peroxisome proliferator‐activated receptor alpha (PPARα), activates the PPARα/carnitine palmitoyltransferase 1A (CPT1A) signaling axis. This molecular interaction upregulates the expression of acyl‐CoA oxidase 1 (Acox1), a key enzyme in peroxisomal fatty acid oxidation, thereby enhancing mitochondrial β‐oxidation capacity and promoting the catabolism of fatty acids (Li et al. 2021). Notably, the lipid‐regulating effects of SSD were abrogated by the PPARα inhibitor GW6471, indicating that SSD alleviates MASLD through concurrent modulation of both the PPARα and INSIG/SREBP‐1c pathways. Further mechanistic insights reveal that PPARα, in addition to its direct role in lipid metabolism, also exerts agonistic effects on the INSIG1/2 targets upstream of SREBP1c—a key regulatory node in lipogenesis. This dual targeting of PPARα and INSIG/SREBP‐1c pathways underscores the multitarget mechanism by which SSs improve lipid metabolism disorders in MASLD (Fernández‐Alvarez et al. 2011; Gu et al. 2022). In experiments with high‐fat diet (HFD)‐induced hybrid grouper, SSD was demonstrated to mitigate hepatic lipid accumulation through multiple mechanisms: reducing apoptosis, alleviating oxidative stress, inhibiting lipogenesis, and enhancing lipid oxidation. Furthermore, SSD improved hepatic steatosis by activating the AMPK/PPARα signaling pathway, which upregulated PPARα mRNA expression (Zou et al. 2022).

### 2.2 Ameliorate Insulin Resistance

Insulin resistance (IR) serves as a primary pathogenic driver of MASLD progression. In the liver, the impaired ability of insulin to suppress hepatic glucose production leads to TG accumulation. Additionally, IR modulates hepatic lipid metabolism, inflammatory responses, and fibrogenic processes through multiple molecular mechanisms, thereby exacerbating MASLD progression. Notably, SSs ameliorate MASLD by improving IR, involving coordinated regulation of multiple signaling pathways (Bo et al. 2024; Palma et al. 2022). These experimental findings further confirm that in HFD‐induced Sprague–Dawley (SD) rat models, SSA intervention significantly activates AMPK/PPARα‐related signaling pathways. Concurrently, fasting blood glucose (FBG), fasting insulin (FINS), and the homeostasis model assessment of insulin resistance (HOMA‐IR) index exhibited statistically significant reductions, which effects effectively ameliorated IR in MASLD rats. Mechanistic studies revealed that SSA improves metabolic parameters by dual actions: inhibiting hepatic gluconeogenesis and enhancing glucose uptake in peripheral tissues. These processes collectively led to a marked reduction in HOMA‐IR, restoration of insulin sensitivity, and ultimately, attenuation of MASLD progression (Gu et al. 2021). Additionally, SSD promotes hepatic glucose oxidative metabolism through a dual mechanism: downregulating the adrenergic Receptor Beta 2 (ADRB2) signaling pathway, which in turn inhibits the expression of glycolytic rate‐limiting enzymes hexokinase 2 (HK2) and glucose transporter 1 (GLUT1) in hepatocytes (He et al. 2023). Notably, SSA additionally reduces lactate production by inhibiting the Akt/STAT3 signaling pathway. These combined effects mitigate hepatic glucose output, enhance insulin sensitivity, and further ameliorate IR, thereby halting the progression of MASLD (Y. Zhang et al. 2023).

### 2.3 Inhibit Chronic Inflammation

Chronic inflammation serves as a key driver of liver injury, with chronic low‐grade inflammation—triggered by inflammatory cytokines released by the body—participating in the entire pathophysiological process of MASLD development. Zhu et al.'s study demonstrated that in vitro experiments (using LPS‐stimulated RAW 264.7 macrophages) revealed that SSA inhibited the NF‐κB and MAPK signaling pathways. This inhibition significantly reduced the release and mRNA expression of pro‐inflammatory cytokines (TNF‐α, IL‐1β, IL‐6), suppressed the mRNA and protein expression of inducible nitric oxide synthase (iNOS) and COX‐2, and simultaneously upregulated the expression of the anti‐inflammatory cytokine IL‐10. These findings collectively indicate that SSA exerts potent anti‐inflammatory effects (Zhu et al. 2013). Studies have demonstrated that treatment with SSD at doses of 1–2 mg/kg in a carbon tetrachloride (CCl_4_)‐induced acute liver injury mouse model significantly inhibited NLRP3 inflammasome expression and suppressed downstream inflammatory cascades. Compared with the model group, this intervention reduced the release of pro‐inflammatory cytokines TNF‐α, IL‐1β, and IL‐6, decreased hepatic malondialdehyde (MDA) content, and concurrently elevated superoxide dismutase (SOD) activity and total glutathione (T‐GSH) levels, ultimately improving liver function (Chen et al. 2020). However, other studies have demonstrated that high doses of SSD induce hepatotoxicity, whereas only lower concentrations exhibit significant hepatoprotective activity (Chen et al. 2013; Zhang et al. 2016). Studies have reported that 2 mg/kg SSD primarily protects mice from acetaminophen (APAP)‐induced hepatotoxicity by downregulating NF‐κB and STAT3‐mediated inflammatory signaling (Liu et al. 2014). Collectively, SSs may treat MASLD by ameliorating chronic low‐grade inflammation in the body through the aforementioned mechanisms.

### 2.4 Ameliorate Oxidative Stress and Autophagy

Endoplasmic reticulum stress (ERS) and oxidative stress (OS) are critical drivers of MASLD progression. Imbalances between the endogenous antioxidant defense system and reactive oxygen species (ROS) production lead to oxidative stress‐induced tissue damage. These two processes are closely interconnected—severe oxidative stress can drive disease progression to MASH, activate hepatic stellate cells (HSCs), and ultimately induce hepatic fibrosis (Lazarus et al. 2022). Studies have demonstrated that scavenging ROS, modulating the redox enzyme system, and activating endogenous antioxidant pathways can alleviate oxidative stress‐induced damage, thereby effectively mitigating hepatic steatosis (Ashraf and Sheikh 2015; Lebeaupin et al. 2018). In HFD‐induced mouse models, SSD significantly alleviated oxidative stress by enhancing the activity of antioxidant enzymes (catalase, glutathione peroxidase, and superoxide dismutase) and inhibiting ROS accumulation. Concurrently, SSD downregulated the expression of inflammation‐related genes (NF‐κB, iNOS, COX‐2), suppressed ER stress signaling pathways (reducing levels of phosphorylated eukaryotic initiation factor 2α, activating transcription factor 4, C/EBP homologous protein, and ubiquitin‐binding protein 62), and thereby improved MASLD (Chang et al. 2021). Studies have demonstrated that autophagy is closely associated with hepatic steatosis. Inhibition of autophagy leads to lipid accumulation in hepatocytes, thereby contributing to the development of fatty liver. Conversely, restoration or enhancement of autophagy ameliorates hepatic steatosis (Moschen et al. 2013; Singh et al. 2009). Lee et al. discovered through a combined approach of computational modeling and animal experiments that SSA inhibits inflammatory responses by downregulating the mRNA expression of pro‐inflammatory cytokines TNF‐α and NF‐κB. Concurrently, SSA upregulated the metabolic regulator fibroblast growth factor 21 (FGF21) to promote energy metabolic balance and enhanced the expression of the key autophagy gene ATG7 to improve cellular autophagy function (Lee, Noh, and Lee 2024).

### 2.5 Modulate Gut Microbiota and Metabolites

Growing evidence indicates that the gut microbiota‐liver axis plays a critical role in the pathogenesis of MASLD. The mechanism by which SSs—particularly SSD—ameliorate MASLD through modulation of gut microbiota has been supported by multiple studies. For instance, research has demonstrated that treatment of HFD‐fed mice with SSD increases the abundance of beneficial bacteria (e.g., the *Lactobacillusgenus*) while reducing the relative abundance of obesity‐associated bacterial genera (e.g., *Lachnospiraceae, Roseburia*). These changes further elevate short‐chain fatty acid levels and activate relevant signaling pathways, ultimately ameliorating MASLD via the gut microbiota‐SCFA‐thermogenic adipose axis (Wang, Chen, et al. 2025). Previous studies have confirmed that *Akkermansia muciniphila (AKK)* can inhibit the development of obesity and IR by regulating adipose tissue metabolism (Schneeberger et al. 2015). Studies by Li, Guan, et al. (2017) have confirmed that SSD improves MASLD through the gut microbiota‐bile acid axis. Specifically, SSD inhibits the intestinal farnesoid X receptor (FXR) signaling pathway, significantly increasing the relative abundance of probiotic bacteria AKK and *Bacteroides* while reducing the colonization of conditionally pathogenic bacteria such as *Clostridium scindens* and *Clostridium leptum* . These changes reshape the gut microbiota composition, reduce bile acid reabsorption, and mitigate hepatic lipid deposition and IR. Notably, the enrichment of AKK synergizes with these effects by enhancing mucus layer integrity, reducing intestinal permeability, and dampening systemic inflammation, thereby improving intrahepatic oxidative stress status (Wang, Li, et al. 2023). These mechanisms suggest that SSs, through multi‐target intervention in the gut‐liver axis metabolic network, provide a novel molecular basis for the treatment of MASLD.

### 2.6 Inhibit Fibrosis

**FIGURE 1:**
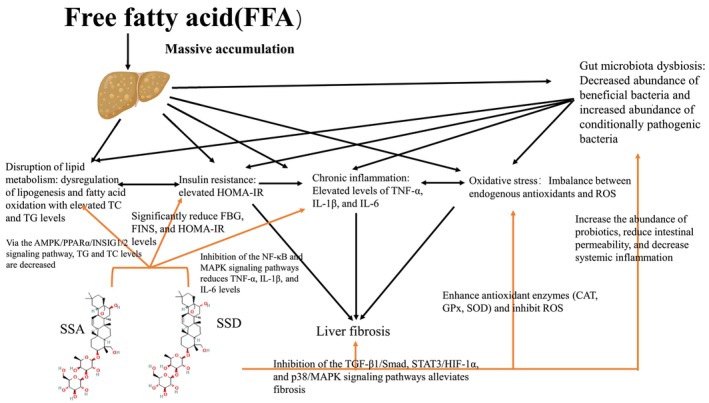
Schematic diagram of the pathological mechanisms induced by massive accumulation of free fatty acids (FFA) and the therapeutic potential of SSA/SSD. Excessive accumulation of FFA triggers a cascade of metabolic and inflammatory disorders, including dysregulated lipid metabolism (characterized by abnormal lipogenesis and fatty acid oxidation, elevated total cholesterol [TC] and triglyceride [TG] levels), insulin resistance (evidenced by increased homeostasis model assessment of insulin resistance [HOMA‐IR], fasting blood glucose [FBG], and fasting insulin [FINS]), chronic inflammation (marked by elevated pro‐inflammatory cytokines TNF‐α, IL‐1β, and IL‐6), oxidative stress (due to an imbalance between endogenous antioxidants and reactive oxygen species [ROS]), and gut microbiota dysbiosis (manifested as reduced beneficial bacteria and increased conditional pathogenic bacteria). These interconnected processes drive liver fibrosis through activation of pro‐fibrotic signaling pathways, such as TGF‐β1/Smad, STAT3/HIF‐1α, and p38/MAPK. This diagram illustrates the complex interplay of metabolic, inflammatory, and fibrotic pathways in FFA‐related disorders and highlights the multi‐target therapeutic effects of SSA and SSD.

MASLD has an insidious onset, with persistent early‐stage hepatic inflammation often remaining asymptomatic until progression to advanced stages—such as liver fibrosis and cirrhosis—where symptoms like liver failure, portal hypertension, or cancer emerge. Studies have reported a prevalence of 28.77% in urban areas of China, among whom 16.87% of MASLD patients exhibit significant liver fibrosis (Hou et al. 2024). Studies have demonstrated that intervention with SSD in rat hepatic stellate cells blocks pro‐fibrotic signaling transmission by inhibiting Smad2/3 phosphorylation downstream of the TGF‐β1/Smad signaling pathway and upregulating the expression of inhibitory Smad7 (Chen et al. 2022). Lin et al.'s study demonstrated that treatment with SSD in vitro cultured rat hepatic stellate cell line hepatic stellate cell line T6 (HSC‐T6) exerts anti‐fibrotic effects via an ERβ‐mediated mechanism. Specifically, SSD inhibits the activation of the p38/MAPK signaling pathway through ERβ‐mediated effects, thereby regulating the expression of downstream matrix metalloproteinase‐1 and tissue inhibitor of matrix metalloproteinase‐1. This modulation promotes extracellular matrix degradation, inhibits HSC‐T6 activation, and ultimately attenuates liver fibrosis (Lin et al. 2016). SSB1 can directly bind to the S319 residue in the coiled‐coil domain of STAT3. It not only reduces the transcriptional activity of STAT3, but also blocks the interaction between STAT3 and Gli1 and promotes the degradation of Gli1 via the ubiquitin‐proteasome system, thereby inducing the apoptosis of activated hepatic stellate cells and reducing the deposition of extracellular matrix. Furthermore, experiments have confirmed that the antifibrotic effect of SSB1 completely disappears when STAT3 is knocked down, verifying that SSB1 alleviates liver fibrosis (Shao et al. 2025). This paper summarizes recent research on SSs and describes their model method, animal, dose, period, and effects (Table 1). Figure 1 summarizes the pathological mechanisms induced by massive accumulation of free fatty acids (FFA) and the therapeutic potential of SSA/SSD.

**TABLE 1:** Mechanisms of action of SSs in the treatment of liver disease.

Model method	Animal	Route of administration	Sex	Group sizes	Randomization	Bupleurinate type	Dose	Period	Results	References
High‐fat diet induction: 73% normal chow, 20% lard, 4% white sugar, 2% milk powder, 1% cholesterol	Sprague–Dawley rats	Normal saline/SSA: oral gavage GW6471: intraperitoneal injection	Male	Control group: 10 rats NAFLD group: 12 rats—SSA intervention group: 12 rats—SSA + GW6471 intervention group: 12 rats	Random grouping mentioned; specific method not specified	SSA	Control group: equal volume normal saline (oral gavage + intraperitoneal injection, once daily, intervention phase). NAFLD group: equal volume normal saline (oral gavage + intraperitoneal injection, once daily, intervention phase). SSA intervention group: 5 mg kg^−1^ day^−1^ (dissolved in normal saline, oral gavage). SSA + GW6471 group: SSA 5 mg kg^−1^ day^−1^ (oral gavage) + GW6471 3.5 mg kg^−1^ day^−1^ (intraperitoneal injection)	Modeling phase: 8 weeks—Intervention phase: 14 days—total period: 8 weeks + 14 days	Serum biomarkers: FBG (mmol/L), FINS (mU/L), HOMA‐IR significantly higher in NAFLD group vs. control (all *p* < 0.05); SSA reduced these indicators vs. NAFLD group (*p* < 0.05); SSA + GW6471 reversed SSA's effects (*p* < 0.05). Hepatic indicators: Liver index (%), hepatic TC (mmol/g), TG (mmol/g), FFA (mmol/g) elevated in NAFLD group (*p* < 0.05); SSA reduced these parameters (*p* < 0.05); SSA + GW6471 attenuated SSA's effects (*p* < 0.05). Histology & protein: NAFLD group showed abundant lipid droplets, reduced PPARα (relative expression) and p‐AMPK/AMPK ratio (*p* < 0.05); SSA improved histology and upregulated PPARα/p‐AMPK (*p* < 0.05); SSA + GW6471 counteracted these effects (*p* < 0.05)	(Gu et al. 2021)
High‐fat diet (42% kcal from fat, 0.2% cholesterol) + glucose‐fructose water (18.9 g/L D‐glucose + 23.1 g/L D‐fructose)	8‐week‐old C57BL/6J mice	Oral gavage (all interventions)	Male	Negative control group: 6 mice—HFSW group: 6 mice—HFSW + SSD (5 mg/kg) group: 6 mice—HFSW + SSD (10 mg/kg) group: 6 mice—HFSW + SSD (20 mg/kg) group: 6 mice—HFSW + Simvastatin (5 mg/kg, positive control) group: 6 mice	Random grouping mentioned; specific method not specified	SSD	Negative control/HFSW group: Equal volume solvent (oral gavage). HFSW + SSD groups: 5/10/20 mg kg^−1^ day^−1^ (oral gavage). HFSW + Simvastatin group: 5 mg kg^−1^ day^−1^ (oral gavage)	Total period: 8 weeks—Modeling phase: 8 weeks (HFSW feeding for groups 2–6; normal chow for control)—Intervention phase: 4 weeks (drug gavage for groups 3–6; solvent for control/HFSW group)	Hepatic steatosis: SSD reduced lipid droplets (Oil Red O staining, relative area) dose‐dependently, decreased liver index (%), with superior efficacy vs. Simvastatin. Adipose tissue: SSD reduced WAT weight (g)/adipocyte size (μm^2^), reversed BAT “whitening” (relative area), and increased BAT thermogenesis (body temperature, ° C). Molecular mechanism: SSD activated PPARα (luciferase activity, relative value), upregulated INSIG1/2 (mRNA relative expression), inhibited SREBP1c maturation (protein relative expression), reduced FASN/ACACA (mRNA relative expression), promoted Acox1/Cpt1α (mRNA relative expression); GW6471 blocked these effects. Positive control: Simvastatin improved steatosis but less effective than high‐dose SSD	(Gu et al. 2022)
In vitro: 20% lipid emulsion (LE, 2 mL/L)‐induced primary hepatocyte steatosis. In vivo: High‐fat diet (crude lipid: 15.22% ± 0.59%, composed of 44% fish meal, 19% soybean meal, 20% flour, 5% soybean oil +5% fish oil)	In vitro: Primary hepatocytes of hybrid grouper ( *Epinephelus lanceolatus* ♂× *Epinephelus fuscoguttatus* ♀). In vivo: Juvenile hybrid groupers (initial body weight: 25.58 ± 0.5 g)	In vitro: LE/SSD added to culture medium. In vivo: SSD mixed into high‐fat diet (oral administration, twice daily)	Not specified (presumed mixed sex)	In vitro (3–5 replicates/group): Control, Model (LE‐treated), Recovery (LE + normal medium), SSD 100/200/400 ng/mL groups. In vivo (300 fish, 15 cages): 5 groups (SSD‐0/100/200/400/800), 3 replicate cages/group, 20 fish/cage	In vitro: random assignment of isolated hepatocytes. In vivo: random division of fish into groups; random cage positioning	SSD	In vitro: LE 2 mL/L; SSD 100/200/400 ng/mL. In vivo: SSD supplementation: 0 (SSD‐0), 100 (SSD‐100), 200 (SSD‐200), 400 (SSD‐400), 800 (SSD‐800) mg/kg diet	In vitro: total period 72 h (48 h seeding + 48 h LE induction + 24 h SSD treatment). In vivo: acclimation 2 weeks + formal feeding 56 days—total period: 58 days	In vitro: SSD (100–400 ng/mL) increased LE‐induced hepatocyte viability (%) (*p* < 0.05), enhanced CAT/SOD/GSH‐Px activities (U/mg prot) (*p* < 0.05), reduced vacuoles/lipid droplets (relative area), downregulated G6PD/ME1/FAS (mRNA relative expression), upregulated AMPKα/PPARα/CPT1 (mRNA relative expression) (*p* < 0.05). In vivo: SSD‐200/400 groups showed higher final body weight (g)/weight gain rate (%) vs. control (*p* < 0.05); SSD‐100‐400 groups reduced hepatosomatic index (%) and lipid droplets (relative area) (*p* < 0.05); SSD‐800 group induced hepatic necrosis; SSD‐200/400 upregulated AMPKα/PPARα/CPT1, downregulated FAS (mRNA relative expression) (*p* < 0.05)	(Zou et al. 2022)
LPS ( *Escherichia coli* O26:B6)‐stimulated inflammatory cell model	Mouse macrophage cell line RAW 264.7	In vitro: SSA/LPS added to culture medium; SSA pretreated for 1 h before LPS stimulation	Not applicable (in vitro cell line)	7 groups (3–6 replicates/group): Control group (LPS 1 mg/L, no SSA)—SSA 3.125/6.25/12.5/25/50/100 μM groups	Random assignment of cells after seeding (consistent initial density)	SSA	SSA: 3.125/6.25/12.5/25/50/100 μM (1 h pretreatment). LPS: 1 mg/L (1 μg/mL), stimulated for 18 h (MTT/ELISA/Western blot) or 3 h (real‐time PCR)	Cell acclimation: 24 h after seeding—SSA pretreatment: 1 h—LPS stimulation: 3/18 h—total period: 28–43 h	Cytotoxicity: 12.5–100 μM SSA reduced LPS‐stimulated cell viability (%) (*p* < 0.05); 3.125/6.25 μM showed no cytotoxicity. Inflammatory cytokines: SSA dose‐dependently inhibited LPS‐induced TNF‐α/IL‐1β/IL‐6 release (pg/mL, ELISA) and mRNA expression (relative value, real‐time PCR) (*p* < 0.05), upregulated IL‐10 mRNA (relative value) (*p* < 0.05). Inflammatory mediators: SSA reduced LPS‐induced iNOS/COX‐2 mRNA (relative value) and protein (relative expression) (*p* < 0.05). Signaling pathways: SSA inhibited NF‐κB (reduced IκBα phosphorylation/protein relative expression, p65 nuclear translocation/relative area) and MAPK (reduced p38 MAPK/c‐JNK phosphorylation/protein relative expression) (*p* < 0.05)	(Zhu et al. 2013)
5% carbon tetrachloride (CCl_4_, dissolved in olive oil)‐induced acute liver injury	7–9‐week‐old ICR mice (body weight: 23–28 g)	CCl_4_: intraperitoneal injection (single dose). SSD/mito‐Tempo: intraperitoneal injection (pretreated at 24 and 0.5 h before CCl_4_)	Male	7 groups, 7 mice/group:—Control group—0.2 mg/kg SSD alone group—CCl_4_ model group—CCl_4_ + 1.0/1.5/2.0 mg/kg SSD groups—CCl_4_ + 3 mg/kg mito‐Tempo group (positive control)	Random assignment after 1‐week acclimation (consistent initial body weight/health status)	SSD	CCl_4_: 5% concentration, 0.25 mL/kg (single intraperitoneal injection). SSD: 0.2 mg/kg (alone group); 1.0/1.5/2.0 mg/kg (intervention groups, intraperitoneal injection).\ mito‐Tempo: 3 mg/kg (intraperitoneal injection, positive control)	Acclimation: 1 week—treatment: 1 day—detection: 24 h after CCl_4_ injection—total period: ~8 days	Liver injury: SSD (1.0–2.0 mg/kg) dose‐dependently reduced centrilobular necrosis (relative area), decreased serum ALT/AST/LDH (U/L) (*p* < 0.05/*p* < 0.01), comparable to mito‐Tempo. Oxidative stress: SSD (1.5–2.0 mg/kg) reduced hepatic MDA (nmol/mg prot)/mitochondrial superoxide (relative fluorescence intensity), increased SOD/GPx/CAT activities (U/mg prot) (*p* < 0.05/*p* < 0.01). NLRP3 inflammasome: SSD downregulated NLRP3/ASC/Caspase 1 mRNA (relative value)/protein (relative expression), reduced IL‐1β/IL‐18 secretion (pg/mL) (*p* < 0.05/*p* < 0.01). Mitochondrial ROS: SSD dose‐dependently decreased CCl_4_‐induced mitochondrial ROS (relative fluorescence intensity)	(Chen et al. 2020)
Acetaminophen (APAP)‐induced acute liver injury	6–7‐week‐old C57BL/6J mice	SSD: intraperitoneal injection (once daily for 5 consecutive days). APAP: intraperitoneal injection (single dose, 30 min after last SSD injection)	Male	4 groups: Vehicle/control group—SSD/control group—Vehicle/APAP group—SSD/APAP group	Random division (consistent initial body weight/health status)	SSD	SSD: 2 mg kg^−1^ day^−1^ (dissolved in normal saline with 0.1% Tween 20, intraperitoneal injection). APAP: 200 mg/kg (dissolved in warm normal saline, 20 mg/mL, single intraperitoneal injection). Solvent control: equal volume normal saline with 0.1% Tween 20/warm normal saline	Acclimation: 1–3 days—SSD pretreatment: 5 consecutive days—APAP induction: 30 min after last SSD injection—detection: 4/24 h after APAP injection—total period: ~6 days	Hepatoprotection: SSD/APAP group had lower serum ALT/AST (U/L) vs. vehicle/APAP group; HE staining showed no obvious liver damage (relative area) vs. extensive central venous necrosis in vehicle/APAP group. Inflammatory signaling: SSD reduced hepatic NF‐κB p65/STAT3 phosphorylation (protein relative expression) (*p* < 0.01), reversed APAP‐induced upregulation of Il6/Ccl2/Socs3/Fga/Fgb/Fgg mRNA (relative value), upregulated Il10 mRNA (relative value) (*p* < 0.05/*p* < 0.01). Apoptosis: SSD upregulated Bcl‐2 mRNA (relative value), downregulated Bax mRNA (relative value) (*p* < 0.05). Irrelevant mechanism: no effect on CYP2E1/CYP3A11, SOD1/SOD2 (mRNA relative expression), GSH (mg/g prot), or PPARα pathway (protein relative expression)	(Liu et al. 2014)
TAA‐induced liver injury: Intraperitoneal injection of 100 mg/kg TAA for 6 weeks. HFD‐induced NAFLD: High‐fat diet (5.16 kcal/g, 31.66% lard) for 10 weeks	5‐week‐old male C57BL/6J mice (initial body weight: 18–20 g)	TAA: intraperitoneal injection. SSD: oral gavage. HFD: ad libitum feeding	Male	Total 40 mice: TAA‐induced liver injury (*n* = 24): Control, TAA, TAA + SSD groups (8 mice/group). HFD‐induced NAFLD (*n* = 16): HFD control, HFD + SSD groups (8 mice/group)	Random assignment (consistent initial body weight/health status)	SSD	TAA: 100 mg/kg (intraperitoneal injection, 6 consecutive weeks). SSD: 2 mg kg^−1^ day^−1^ (dissolved in normal saline, oral gavage, 8 consecutive weeks). HFD: 5.16 kcal/g (31.66% lard, 1.12 μg/g chromium, diet 58Y1), ad libitum feeding for 10 weeks. Normal diet: 3.3 kcal/g (acclimation/control groups)	Acclimation: 4 weeks—Model induction: 6 weeks (TAA) / 10 weeks (HFD)—Drug intervention: 8 weeks—Total period: 18 weeks (TAA model)/22 weeks (NAFLD model)	TAA‐induced liver injury: SSD increased body weight/food intake/food efficiency (*p* < 0.05), reduced serum ALT/AST/ALP/γ‐GT/bilirubin (*p* < 0.05), decreased IL‐1β/TNF‐α/CRP/COX‐2/NF‐κB/iNOS (*p* < 0.05), increased FGF21/CAT/GPx/SOD (*p* < 0.05), improved histology (reduced necrosis/swelling, lower Suzuki score). HFD‐induced NAFLD: SSD reduced body weight/weight gain/hepatic steatosis score (*p* < 0.05), decreased serum ALT/AST/triglycerides (serum/hepatic) (*p* < 0.05), downregulated FABP4/SREBP1/ER stress proteins/p62 (*p* < 0.05)	(Chang et al. 2021)
HFD‐induced obesity and insulin resistance (HFD: 60% kcal from fat; normal chow: 10% kcal from fat)	6‐week‐old C57BL/6N mice	SSA/Atorvastatin: oral gavage (once daily). Diet: ad libitum feeding	Male	20 mice, 5 mice/group: NC group (normal chow + normal saline)—HFD group (high‐fat diet + normal saline)—HFD + ATO group (HFD + 10 mg/kg day^−1^ atorvastatin)—HFD + SSA group (HFD + 10 mg/kg day^−1^ SSA)	Random assignment after acclimation: 5 mice for NC group; 15 mice randomly divided into HFD/ATO/SSA groups (consistent initial body weight)	SSA	SSA: 10 mg kg^−1^ day^−1^ (dissolved in normal saline, oral gavage, 9 consecutive weeks). Atorvastatin (ATO): 10 mg kg^−1^ day^−1^ (dissolved in normal saline, oral gavage, 9 consecutive weeks, positive control). Diet: NC (D12450B, 10% kcal from fat); HFD (D12492, 60% kcal from fat), ad libitum feeding for 14 weeks	Acclimation: 1 week—Model induction + diet: 14 weeks (HFD for HFD/ATO/SSA groups; NC for control)—Drug intervention: Weeks 6–14 (9 weeks)—Detection: OGTT (Week 11), OFTT (Week 12), sacrifice (Week 14)	Body weight/organs: liver weight (g) lower in SSA group vs. HFD group (1.05 ± 0.20 vs. 2.48 ± 0.19, *p* < 0.001); no difference in epididymal fat weight (g). Glucose metabolism: reduced OGTT 30/60 min blood glucose (mg/dL), OGTT AUC (mg min/dL), fasting glucose (mg/dL)/insulin (ng/mL)/HOMA‐IR (*p* < 0.05). Lipid metabolism: decreased TC/LDL‐C/NEFA (mg/dL/mEq/L), increased HDL‐C (mg/dL), reduced OFTT 120 min triglycerides (mg/dL)/OFTT AUC (mg·min/dL) (*p* < 0.05/*p* < 0.001). Liver function: lower AST/ALT (U/L) vs. HFD group (*p* < 0.05). Kupffer cells: reduced KCs proportion (%), increased M2‐type (CD206+) (%), decreased M1‐type (CD11c+) (%) (*p* < 0.05). Gene expression: downregulated TNF‐α/NF‐κB p65/F4/80 (mRNA relative expression), upregulated ATG7/FGF21 (mRNA relative expression) (*p* < 0.05/*p* < 0.01). Histology: reduced hepatic fat area (%) (24.03 ± 2.21 vs. 39.34 ± 2.55, *p* < 0.001) and epididymal adipocyte area (μm^2^) (*p* < 0.001)	(Lee, Noh, and Lee 2024, p.4)
HFD‐induced obesity, glycolipid metabolism abnormalities, and gut microbiota dysbiosis (HFD: 60% kcal from fat; normal diet: 10% kcal from fat)	5‐week‐old C57BL/6J mice	SSD/Miglitol: oral gavage (once daily). Diet: ad libitum feeding	Male	40 mice, 10 mice/group: ND group (normal diet + normal saline)—HFD group (high‐fat diet + normal saline)—HFD + SSD group (HFD + 20 mg/kg day^−1^ SSD). HFD + MI group (HFD + 5 mg/kg day^−1^ miglitol, positive control)	Random assignment after 1‐week acclimation (consistent initial body weight/health status); random order for metabolic cage detection/tissue sampling	SSD	SSD: 20 mg kg^−1^ day^−1^ (dissolved in normal saline, oral gavage, 12 consecutive weeks). Miglitol (MI): 5 mg kg^−1^ day^−1^ (dissolved in normal saline, oral gavage, 12 consecutive weeks, positive control). Diet: ND (10% kcal from fat); HFD (60% kcal from fat), ad libitum feeding for 12 weeks	Acclimation: 1 week—Treatment + diet: 12 weeks (HFD for HFD/SSD/MI groups; ND for control)—Detection: OGTT (Week 11), energy metabolism (CLAMS‐12), cold‐stimulated thermography (Weeks 11–12), sacrifice (Week 12)	Body weight/tissues: SSD inhibited weight gain (g) (*p* < 0.05), reduced liver/eWAT/pWAT/iWAT weight (g) (*p* < 0.05); no effect on food intake (g)/spleen/kidney weight (g). Glycolipid metabolism: decreased serum TG/TC/LDL‐C (mmol/L), increased HDL‐C (mmol/L), reduced fasting blood glucose (mmol/L)/OGTT AUC (mmol·min/L) (*p* < 0.05). Energy expenditure: Higher VO_2_/VCO_2_/EE (mL/kg/min/kcal/kg/h) vs. HFD group (*p* < 0.05); increased interscapular skin temperature (°C) after cold stimulation (34.41 vs. 30.86, *p* < 0.05). Thermogenic fat activation: Upregulated UCP1/SIRT1/PGC‐1α (mRNA/protein relative expression) in BAT/iWAT, activated PKA‐AMPK/p38 pathway (phosphorylation/protein relative expression) (*p* < 0.05). Gut microbiota: reversed HFD‐induced dysbiosis, decreased *Faecalibaculum*/unidentified_Lachnospiraceae (relative abundance), increased *Lactobacillus*/*Tyzzerella*/*Bilophila*/*Oscillibacter* (relative abundance) (*p* < 0.05). SCFAs: Increased fecal acetate/propionate/butyrate (mmol/L), upregulated SCFA‐synthesizing enzymes (Ack/Mcd/BCoA/PduP) and receptors (Ffar3/Ffar2/Hcar2) (mRNA relative expression) (*p* < 0.05)	(Wang, Chen, et al. 2025)
H_2_O_2_‐induced oxidative stress in hepatic stellate cells (activates TGFβ‐Smad pathway)	Rat hepatic stellate cell line HSC‐T6	In vitro: SSD/E_2_/estrogen receptor antagonists/H_2_O_2_ added to culture medium (37°C, 5% CO_2_ incubator)	Not applicable (in vitro cell line)	4 basic groups × 4 subgroups = 96 experimental wells (6 replicates/subgroup):—Blank basic group—ICI182780 basic group (ER complete antagonist)—MPP basic group (ERα‐specific antagonist)—(R,R)‐THC basic group (ERβ‐specific antagonist). Subgroups: Control, Model, SSD, E_2_	Random seeding (consistent initial density: 1 × 10^4^ cells/100 μL); random assignment to groups after 24 h adhesion	SSD	SSD: 5 μM (added to medium, 24 h treatment). Estradiol (E_2_, positive control): 1 μM (24 h treatment). Estrogen receptor antagonists: ICI182780/MPP/ (R,R)‐THC (1 μM each, 24 h treatment). H_2_O_2_: Added to medium (4 h oxidative stress induction)	Cell adhesion: 24 h after seeding. Drug treatment: 24 h (SSD/E_2_/antagonists). Oxidative stress induction: 4 h (H_2_O_2_). Total period: 52 h	Model validation: TGFβ₁/Smad3 mRNA elevated, Smad7 mRNA reduced in Model group vs. control (*p* < 0.05). SSD effect: TGFβ₁/Smad3 mRNA reduced, Smad7 mRNA increased in SSD group vs. Model group (*p* < 0.05), similar to E_2_ group. ER dependence: ICI182780 and (R,R)‐THC blocked SSD's effects (TGFβ₁/Smad3 rebounded, Smad7 decreased, *p* < 0.05); MPP had no blocking effect (*p* > 0.05)	(Chen et al. 2022)
Activated hepatic stellate cell model (in vitro): Rat hepatic stellate cell line HSC‐T6 (activated phenotype)	Rat hepatic stellate cell line HSC‐T6 (activated phenotype)	In vitro: SSD/E_2_/estrogen receptor antagonists/P38 inhibitor added to culture medium	Not applicable (in vitro cell line)	15 treatment groups (≥ 3 independent experiments/group, 3 replicate wells/group): 5 basic groups: blank control, positive control, ICI‐182.780, MPP, (R,R)‐THC + P38 inhibitor. 3 subgroups: Control, SSD, E_2_	Random seeding (consistent initial density: 1 × 10^4^ cells/well for 96‐well plates; 5 × 10^5^ cells/well for 6‐well plates); random treatment sequence	SSD	SSD: 5 μmol/L (24 h incubation). E_2_ (positive control): 1 μmol/L (24 h incubation). Estrogen receptor antagonists: ICI‐182.780/MPP/ (R,R)‐THC (1 μmol/L each, 1 h pretreatment). P38 MAPK antagonist (SB203580): 50 μmol/L (1 h pretreatment). Control: equal volume medium/solvent	Cell adhesion: 24 h after seeding. Antagonist pretreatment: 1 h. Drug incubation: 24 h. Detection: supernatant/lysate collection for ELISA/Western blot. Total period: 49 h	ECM metabolism: SSD/E_2_ groups reduced COL‐1 (SSD: 140.95 ± 12.14 ng/mL vs. control: 198.98 ± 15.08 ng/mL) and TIMP‐1 (SSD: 0.23 ± 0.01 vs. control: 0.31 ± 0.01 μmol/L), increased MMP‐1 (SSD: 0.0127 ± 0.0008 vs. control: 0.0049 ± 0.0001 μmol/L) (all *p* < 0.01). P38/MAPK pathway: SSD/E_2_ reduced P‐P38 protein (SSD: 0.51 ± 0.14 vs. control: 1.00 ± 0.11, *p* < 0.01). Antagonist effect: ICI‐182.780/(R,R)‐THC blocked SSD/E_2_'s effects (*p* < 0.01); MPP had no effect (*p* > 0.05). P38 inhibitor: SB203580 increased MMP‐1, decreased TIMP‐1 (*p* < 0.05/*p* < 0.01)	(Lin et al. 2016)
In vitro: TGF‐β1 (10 ng/mL)‐activated hepatic stellate cells (HSC‐T6/LX‐2). In vivo: TAA‐induced (2 weeks) / CCl_4_‐induced (3 weeks) liver fibrosis; STAT3 KD model (AAV8‐ApoE‐shSTAT3, 3 weeks)	Cell lines: Rat HSC‐T6, human LX‐2. Mice: BALB/c mice	In vitro: drugs/plasmids added to culture medium. In vivo: CCl_4_ (intraperitoneal injection, twice weekly); SSB1/S3I‐201 (oral gavage, once daily); AAV8‐ApoE‐shSTAT3 (tail vein injection, single dose)	Cell experiments: not applicable. Mice: not specified	In vitro: ≥ 3 independent replicates/group, 3 replicate wells/group (1 × 10^4^ cells/well for 96‐well plates; 5 × 10^5^ cells/well for 6‐well plates). In vivo: 6 mice/group: Normal, Model (TAA/CCl_4_), SSB1, S3I‐201, STAT3 KD groups	In vitro: random cell seeding (consistent density). In vivo: random assignment after 1‐week acclimation (consistent initial body weight/health status)	SSB1	In vitro: SSB1 1/5/10 μM (1 h pretreatment +24 h TGF‐β1 co‐incubation); TGF‐β1 10 ng/mL; S3I‐201 (STAT3 inhibitor) 50 μM (1 h pretreatment). In vivo: SSB1 10 mg kg^−1^ day^−1^ (oral gavage, 2–4 weeks); S3I‐201 5/10/20 mg kg^−1^ day^−1^ (oral gavage, 2 weeks); CCl_4_ 10% in olive oil 0.5 μL/g (intraperitoneal injection, twice weekly, 3 weeks); AAV8‐ApoE‐shSTAT3 (routine titer, single tail vein injection)	In vitro: 24 h cell adhesion + 1 h pretreatment + 24 h TGF‐β1 stimulation (total 49 h). In vivo: TAA model (2 weeks induction + 2 weeks administration); CCl_4_ model (3 weeks induction + 4 weeks administration); STAT3 KD model (3 weeks knockdown + 3 weeks induction + 4 weeks administration)	In vitro anti‐HSC activation: SSB1 (5/10 μM) dose‐dependently reduced α‐SMA/Collagen I/FN/Vimentin (protein relative expression) (*p* < 0.01); bound STAT3 S319 residue, inhibited Tyr705 phosphorylation (protein relative expression)/dimerization/nuclear translocation (relative area) (*p* < 0.01); blocked STAT3/Gli1 interaction (relative binding affinity), promoted Gli1 degradation (protein half‐life, h), downregulated Bcl2 (mRNA relative expression), induced HSC apoptosis (%) (*p* < 0.01). In vivo anti‐liver fibrosis: SSB1 (10 mg/kg) reduced collagen deposition (Masson/Sirius Red, relative area), decreased liver index (%)/serum ALT/AST/TBIL (U/L/μmol/L) (*p* < 0.01); downregulated α‐SMA/Collagen I/FN (protein relative expression), inhibited STAT3 phosphorylation/Gli1 (protein relative expression) (*p* < 0.01); STAT3 knockdown abolished SSB1's effects	(Shao et al. 2025)

## 3 Advances in Clinical Compound Formulations of Saikosaponins

Although the aforementioned Chinese medicine monomers (SSA and SSD) have demonstrated lipid‐regulating and anti‐fibrotic activities in basic research. But compound Chinese medicines remain the primary choice for clinical application at present. Among them, classic Bupleurum‐containing formulas—including Xiaoyao San (Free and Unfettered Powder), Xiaochaihu Tang (Minor Bupleurum Decoction), and Chaihu Shugan San (Bupleurum Soothing the Liver Powder)—are frequently prescribed for MASLD due to their TCM effects of “soothing the liver, invigorating the spleen, regulating qi, and resolving stagnation.” However, their exact efficacy and advantages still require further verification through more high‐quality clinical studies, such as large‐sample randomized controlled trials (RCTs).

First recorded in Taiping Huimin Heji Ju Fang (Formulary of the Peaceful Benevolent Dispensary), Xiaoyao San consists of 8 herbs: Bupleurum chinense DC., Angelica sinensis (Oliv.) Diels, *Paeonia lactiflora* Pall., Poria cocos (Schw.) Wolf, Atractylodes macrocephala Koidz., processed Glycyrrhiza uralensis Fisch. var. inflata Bat., Mentha haplocalyx Briq., and *Zingiber officinale* Roscoe (Meng et al. 2025). In recent years, several clinical studies have explored the auxiliary therapeutic value of its combination with Western medicine for MASLD. Huang et al. observed through a small‐sample randomized controlled trial (RCT) that after 3 months of treating MASLD patients with modified Xiaoyao San combined with conventional Western medicines (magnesium isoglycyrrhizinate and bicyclol), the improvements in body mass index (BMI) and biochemical indicators in the observation group were more significant than those in the control group; however, this study requires further verification through larger‐sample, multi‐center long‐term follow‐up (Huang and Shi 2019). Similarly, Li's single medium‐sample RCT further found that among 98 patients, after 6 months of treatment with *Xiaoyao San* combined with simvastatin, the improvements in liver function indicators (ALT, AST) and lipid indicators (TC, TG, etc.) were significantly better than those in the group treated with simvastatin alone, with no increase in the incidence of adverse reactions (Li 2019). A single‐center RCT conducted by Qiao et al. showed that when Xiaoyao San combined with a lipid‐regulating prescription was used together with conventional Western medicine to treat MASLD patients with liver depression and spleen deficiency syndrome, the liver function and lipid levels in the observation group were superior to those in the control group, and the serum levels of leptin (LEP), lipopolysaccharide (LPS), and nesfatin‐1 (NSF‐1) also decreased significantly. These findings suggest that Xiaoyao San combined with the lipid‐regulating prescription may play a role in improving relevant indicators in this type of MASLD patients; however, since it was only a single medium‐sample study that did not include gold‐standard indicators such as liver biopsy, nor did it involve the evaluation of long‐term efficacy and cross‐organ effects, it cannot be concluded that it can “effectively treat MASLD patients” based on this, and its clinical value still requires further verification through more high‐quality studies (Qiao et al. 2022). Current studies indicate that Xiaoyao San, either used alone or in combination with Western medicine, may have potential in improving clinical indicators of MASLD, but its efficacy still needs to be verified by large‐sample, rigorously designed RCTs.

Derived from Zhang Zhongjing's Shanghan Zabing Lun (Treatise on Febrile Diseases and Miscellaneous Diseases), Xiaochaihu Tang comprises 7 herbs: Bupleurum chinense DC., Scutellaria baicalensis Georgi, *Panax ginseng* C. A. Mey., *Pinellia ternata* (Thunb.) Breit., processed Glycyrrhiza uralensis Fisch., *Zingiber officinale* Roscoe, and *Ziziphus jujuba* Mill. (Shi et al. 2025). Recent studies have demonstrated its significant efficacy in treating MASLD. A small‐sample RCT involving 64 patients conducted by Tong et al. showed that after 4 weeks of treating MASLD with modified *Xiaochaihu Tang* alone (1 dose per day, decocted to obtain 300 mL), the observation group had better improvements in indicators such as ALT, AST, TG, and HDL‐C than the control group treated with Essentiale Capsules; the proportion of patients achieving “clinical control + marked effect + effective” was 87.5% in the observation group versus 78.1% in the control group, with a statistically significant difference between the two groups (*p* < 0.05) (Tong 2017). Shi Huijie further expanded the sample size to 167 cases and conducted a non‐randomized controlled study: the control group was treated with Silybin Capsules alone (105 mg per dose, 3 times a day), while the study group received Silybin Capsules combined with Xiao Chaihu Decoction (150 mL decoction, 2 times a day); after 12 weeks of treatment, the proportion of patients in the study group meeting the preset lipid improvement criteria (TC reduction ≥ 10% and TG reduction ≥ 20%) was 97.62%, higher than 86.75% in the control group, and the improvements in metabolic indicators and liver function indicators were more significant. However, this study adopted a non‐randomized controlled design without randomization or blinding, which may have selection bias and performance bias, and the rationality of its wide application still needs further verification by high‐quality studies with large samples, randomization, and long‐term follow‐up (Shi 2019). Similarly, a clinical RCT found that after 12 weeks of treatment with modified Xiaochaihu Tang combined with lifestyle intervention, the number of patients with BMI > 25.0 decreased, TG and TC levels decreased, Adiponectin (ADP) level increased, and controlled attenuation parameter (CAP) value decreased; these results indicate that modified Xiaochaihu Tang may help alleviate lipid metabolism disorders and improve hepatic steatosis in MASLD patients (Li, Zhang, and Li 2017). Current evidence suggests that Xiaochaihu Tang may play a role in regulating metabolism and inflammatory responses, but its long‐term efficacy and safety in MASLD still require further verification.

First documented in Jingyue Quanshu (Complete Works of Zhang Jingyue), Chaihu Shugan San includes 7 herbs: *Citrus reticulata* Blanco, Bupleurum chinense DC., Ligusticum chuanxiong Hort., *Cyperus rotundus* L., Citrus aurantium L., Paeonia lactiflora Pall., and processed Glycyrrhiza uralensis Fisch., with TCM effects of soothing the liver and regulating the spleen (Li, Guan, et al. 2025). Multiple studies have reported its significant efficacy in treating MASLD, with mechanisms spanning multi‐target actions such as metabolic regulation, anti‐inflammation, improvement of insulin sensitivity, and gut microbiota modulation. A randomized controlled trial by Zhu et al. found that compared with the control group treated with Polyene Phosphatidylcholine Capsules, after 4 months of treatment with Chaihu Shugan San, the observation group showed decreased levels of TC, TG, and LDL‐C, increased HDL‐C, and significantly reduced liver function indicators (ALT, AST) compared with pre‐treatment (*p* < 0.05) (Zhu et al. 2018). A study by He et al. included 120 patients with MASLD; after randomization, the observation group received Chaihu Shugan San for 60 days, and the proportion of patients meeting the preset efficacy criteria (based on improvements in liver function, lipid metabolism, etc.) was 80.70%, slightly higher than that of the control group, with more significant effects in regulating FGF21 and NSF‐1 levels and improving IR (*p* < 0.05) (He et al. 2022). In a randomized controlled trial conducted by Su Wei, patients in the study group received conventional Western medicines (Polyene Phosphatidylcholine Capsules, Metronidazole Tablets, Taurine Capsules, and intravenous Polymyxin B Sulfate for Injection) combined with Chaihu Shugan San for 2 consecutive months; the proportion of patients meeting the preset efficacy criteria (based on imaging and symptom improvements) was 94.44%, higher than the control group (74.29%), and the improvements in liver function indicators (ALT, AST) and lipid indicators (TG, TC, etc.) were more significant (*p* < 0.05) (Su 2021). Similarly, a single small‐sample randomized controlled trial by Xuan showed that after 8 weeks of treatment with Chaihu Shugan San alone, the overall efficacy rate of the treatment group was 91.11%, slightly higher than the control group (treated with Polyene Phosphatidylcholine Capsules, 82.22%), with more significant improvements in ALT and GGT levels (*p* < 0.05) and no adverse reactions reported (Xuan 2019). Additionally, in a single medium‐sample randomized, single‐blind, placebo‐controlled trial by Xie Weining, it was found that on the basis of lifestyle intervention, after 12 weeks of treatment with Chaihu Shugan San, the total effective rate of MASLD evaluated by color Doppler ultrasound was 81.08%, higher than 68.57% of the control group; moreover, it showed more significant effects in improving liver function (ALT, AST), lipid levels (TC, LDL‐C), inflammatory factors (IL‐6, TNF‐α), and modulating gut microbiota (increasing the abundance of *Bifidobacterium* and *Lactobacillus*, reducing *Enterococcus* and *Enterobacter*) (*p* < 0.05), suggesting that it regulates inflammation and microbiota balance through the “gut‐liver axis” (Xie et al. 2021). Beyond experimental studies, Chang Shuo et al. used bioinformatics approaches such as network pharmacology and molecular docking and found that the active components of Chaihu Shugan San (including saikosaponins) are theoretically associated with the pathological mechanisms of type 2 diabetes mellitus, MASLD, and depression through targets such as IL‐6, TNF, and IL‐1β, as well as signaling pathways including HIF‐1, MAPK, and Toll‐like receptors; it was hypothesized that its mechanism may be related to improving IR, inflammatory responses, and lipid metabolism disorders. However, this study only involved basic mechanism exploration without clinical research, lacking support from high‐quality clinical evidence; thus, it cannot imply that Chaihu Shugan San has definite efficacy in MASLD treatment, and its related effects need further verification through in vivo experiments and clinical studies (Chang et al. 2023). Collectively, Chaihu Shugan San has shown certain potential in the adjuvant treatment of MASLD; it can exert synergistic effects when used alone, or in combination with lifestyle intervention or Western medicines, providing an additional research direction for improving metabolism, anti‐inflammation, insulin sensitivity, and gut microbiota. However, the overall evidence is still dominated by single small‐sample studies, and more high‐quality studies are needed to confirm its clinical value (Table 2).

**TABLE 2:** Summary of human clinical studies on saikosaponins for MASLD treatment.

Total sample size	Sample size calculation basis	Randomization design	Blinding design	Prespecified primary outcome indicators	Safety outcomes	Evidence grading	Risk of bias	Citation
60 cases (30 in Observation Group/30 in Control Group)	Not reported	Random sequence generation: random grouping (specific method not specified)	Not adopted	Core indicators: changes in BMI, ALT, GGT, TG, TC levels; clinical therapeutic efficacy. Judgment criteria: Marked effect: indicators (ALT, TG, TC) within normal range, or ALT reduction > 50%, TG reduction > 40%, TC reduction > 20%. Effective: 30% < ALT reduction ≤ 50%, 20% < TG reduction ≤ 40%, 10% < TC reduction ≤ 20%. Ineffective: ALT reduction < 30%, TG/TC reduction insignificant or no improvement. Total effective rate = (marked effect + effective)/total cases × 100%. Measurement method: BMI calculated from height and weight; biochemical indicators (ALT, GGT, TG, TC) detected by laboratory tests. Measurement time points: before treatment and after 3 months of continuous treatment	Prespecified indicators: not reported. Collection method: not reported. Follow‐up duration: 3 months (continuous treatment period). Results: no safety‐related data (e.g., adverse reactions, abnormal organ function) reported	Level 2b (Oxford 2011 Evidence Grading)	Selection bias (unclear randomization method + no allocation concealment), performance bias (no blinding), measurement bias (no explicit reporting of detection instrument brand/standards), follow‐up bias (no long‐term follow‐up beyond treatment period)	(Huang and Shi 2019)
98 cases (49 in Observation Group/49 in Control Group)	Not reported	Random sequence generation: Random number table method (explicitly stated, no information on whether the sequence was independently generated)	Not adopted	Core indicators: clinical therapeutic efficacy; liver function (ALT, AST); blood lipid indicators (TC, TG, HDL‐C, LDL‐C). Judgment criteria: Marked effect: symptoms and signs significantly improved; liver function and blood lipid indicators returned to normal. Effective: symptoms and signs significantly improved; liver function and blood lipid indicators decreased by > 20% compared with baseline. Ineffective: symptoms and signs unchanged or aggravated; no improvement in liver function and blood lipid indicators. Total effective rate = (marked effect + effective)/total cases × 100%. Measurement method: fasting peripheral venous blood (5 mL) centrifuged at 3000 r/min for 5 min; liver function and blood lipid indicators detected by AU5800 automatic biochemical analyzer (Beckman Coulter, USA). Measurement time points: before intervention and after 6 months of continuous treatment	Prespecified indicators: routine blood test, routine stool test, renal function; adverse reactions during treatment. Collection method: regular monitoring of laboratory indicators during treatment; active recording of adverse reactions reported by patients. Follow‐up duration: 6 months (continuous treatment period). Results: routine blood test, routine stool test, and renal function were normal in both groups before and after treatment; 2 cases (4.08%) of skin rash in the Control Group, 1 case (2.04%) of diarrhea in the Observation Group; no statistically significant difference in adverse reaction rates between the two groups (*p* > 0.05); adverse reactions were controlled after drug withdrawal	Level 2b (Oxford 2011 Evidence Grading)	Selection bias (no allocation concealment), performance bias (no blinding), follow‐up bias (no long‐term follow‐up beyond 6‐month treatment period), detection bias (potential subjectivity in symptom/sign evaluation despite clear efficacy criteria)	(Li 2019)
82 cases (41 in Observation Group/41 in Control Group)	Not reported	Random sequence generation: Random grouping (specific method not specified)	Not adopted	Core indicators: clinical therapeutic efficacy; TCM syndrome score; liver function (ALT, AST, GGT); blood lipid indicators (TC, TG, HDL‐C); serum levels of LEP, LPS, NSF‐1 (Nesfatin‐1). Judgment criteria: Marked effect: clinical symptoms basically disappeared; liver function returned to normal. Effective: clinical symptoms partially improved; liver function improved but not normalized. Ineffective: no significant improvement in symptoms or liver function. Total effective rate = (marked effect + effective)/total cases × 100%. TCM syndrome score: evaluated items include nausea/vomiting, abdominal distension, poor appetite, fatigue, hepatosplenomegaly; each item scored 0–3 points (higher score indicates more severe symptoms). Measurement method: Liver function and blood lipids: fasting venous blood (7 mL) detected by automatic biochemical analyzer. LEP, LPS, NSF‐1: fasting venous blood (7 mL) measured by enzyme‐linked immunosorbent assay (ELISA). 4 Measurement time points: Before treatment and after 2 months of continuous treatment	1 Prespecified indicators: adverse reactions during treatment (no severe adverse reactions predefined). 2 Collection method: active recording of adverse reactions reported by patients during treatment. 3 Follow‐up duration: 2 months (continuous treatment period). 5 Results: no severe adverse reactions in either group; 1 case of diarrhea in the Observation Group, 2 cases of diarrhea +1 case of decreased liver function in the Control Group; no statistically significant difference in adverse reaction rates between the two groups (*p* > 0.05)	Level 2b (Oxford 2011 Evidence Grading)	Selection bias (unclear randomization method + no allocation concealment), performance bias (no blinding), measurement bias (potential subjectivity in TCM syndrome score evaluation), follow‐up bias (no long‐term follow‐up beyond 2‐month treatment period)	(Qiao et al. 2022)
64 cases (32 in Observation Group/32 in Control Group)	Not reported	Random sequence generation: Random number table method (explicitly stated; allocation by visit order mentioned but not conflicting with random number table method; no information on independent sequence generation)	Not adopted	Core indicators: liver function (ALT, AST); blood lipid indicators (TC, TG, LDL‐C, HDL‐C); clinical therapeutic efficacy. Judgment criteria (referenced “Guiding Principles for Clinical Research of New Chinese Medicines”): Clinical control: symptoms disappeared; blood lipids and liver function normalized; liver B‐ultrasound echo normal. Marked effect: symptoms disappeared; TG/TC reduced by 20%–40%; ALT/AST reduced by ≥ 40%. Effective: symptoms relieved; TG/TC reduced by 10%–20%; ALT/AST reduced by 20%–40%; liver B‐ultrasound improved. Ineffective: symptoms unchanged; TG/TC reduced by < 10%; ALT/AST reduced by < 20%. Total effective rate = (clinical control + marked effect + effective)/total cases × 100%. Measurement method: serum liver function and blood lipid indicators detected by laboratory tests (detection instrument not specified). Measurement time points: before treatment and after 4 weeks of continuous treatment	Prespecified indicators: not reported (no adverse reaction monitoring mentioned explicitly). Collection method: not reported. Follow‐up duration: 4 weeks (continuous treatment period). Results: no safety‐related data (e.g., adverse reactions, abnormal organ function) reported	Level 2b (Oxford 2011 Evidence Grading)	Selection bias (no allocation concealment), performance bias (no blinding), measurement bias (detection instrument not specified), follow‐up bias (no long‐term follow‐up beyond 4‐week treatment period), reporting bias (unreported safety outcomes)	(Tong 2017)
167 cases (84 in Study Group/83 in Control Group)	Not reported	Random sequence generation: Not specified (only stated “divided into two groups” without explicit randomization method)	Not adopted	Core indicators: liver function (GGT, AST, ALT); blood lipid indicators (LDL‐C, TG, TC); clinical therapeutic efficacy. Judgment criteria: Marked effect: TC reduced by > 20% and TG reduced by > 30%. Effective: TC reduced by 10%–20% and TG reduced by 20%–30%. Ineffective: TC reduced by < 10% or TG reduced by < 20%. Total effective rate = (marked effect + effective)/total cases × 100%. 3 Measurement method: serum indicators detected by radioimmunoassay (kits purchased from Shanghai Sixin Co. Ltd.); detection instrument not specified. 4 Measurement time points: before treatment and after 12 weeks of continuous treatment	Prespecified indicators: not reported (no adverse reaction monitoring or safety indicator assessment mentioned). Collection method: not reported. Follow‐up duration: 12 weeks (continuous treatment period). Results: no safety‐related data (e.g., adverse reactions, abnormal organ function) reported	Level 3b (Oxford 2011 Evidence Grading)	Selection bias (unclear randomization method + no allocation concealment), performance bias (no blinding), measurement bias (detection instrument not fully specified), follow‐up bias (no long‐term follow‐up beyond 12‐week treatment period), reporting bias (unreported safety outcomes)	(Shi 2019)
84 cases (52 in Treatment Group/32 in Control Group)	Not reported	Random sequence generation: Random number envelope method (allocation by visit order; envelopes with pre‐prepared random number cards, whether envelopes were opaque not specified)	Not adopted	Core indicators: number of patients with BMI > 25.0; blood lipid indicators (TG, TC); adiponectin (ADP); controlled attenuation parameter (CAP). Judgment criteria: no predefined “marked effect/effective” classification; efficacy evaluated by changes in indicators (statistical significance of pre‐ vs. post‐treatment differences and inter‐group differences). Measurement method: BMI: calculated as weight (kg)/height (m)^2^. TG/TC: fasting venous blood (3–5 mL) detected by ABBOTT AEROSET automatic biochemical analyzer (kits from Xiamen Technology Co. Ltd.) ADP: fasting venous blood (10 mL) measured by enzyme‐linked immunosorbent assay (ELISA) using Yantai Aidekang Bioengineering automatic enzyme immunoassay workstation (kits from Shanghai Youyu Biotechnology Co. Ltd.) CAP: Quantitative liver steatosis measured by certified operators, unit: dB/m. 4 Measurement time points: before treatment and after 12 weeks of continuous treatment	Prespecified indicators: not reported (no adverse reaction monitoring or safety indicator assessment mentioned). Collection method: not reported. Follow‐up duration: 12 weeks (continuous treatment period); 2 cases lost to follow‐up (1 in each group). Results: no safety‐related data (e.g., adverse reactions, abnormal organ function) reported	Level 2b (Oxford 2011 Evidence Grading)	Selection bias (unverified allocation concealment + unclear envelope opacity), performance bias (no blinding), follow‐up bias (no long‐term follow‐up beyond 12‐week treatment period), reporting bias (unreported safety outcomes)	(Li, Zhang, and Li 2017)
70 cases (40 in Observation Group/30 in Control Group)	Not reported	Random sequence generation: Allocation by visit order (described as “randomly divided” but based on visit sequence, not true randomization)	Not adopted	Core indicators: blood lipid indicators (TC, TG, HDL‐C, LDL‐C); liver function (ALT, AST); liver B‐ultrasound findings. Judgment criteria: no predefined “marked effect/effective” classification; efficacy evaluated by statistical significance of pre‐ vs. post‐treatment indicator changes and inter‐group differences. Measurement method: blood lipid and liver function indicators detected by laboratory tests (detection instrument/kit not specified); liver B‐ultrasound performed but no specific grading standards reported. Measurement time points: before treatment and after 2 courses of treatment (4 months total; 2 months per course)	Prespecified indicators: not reported (no adverse reaction monitoring or safety indicator assessment mentioned). Collection method: not reported. Follow‐up duration: 4 months (2 courses of treatment). Results: no safety‐related data (e.g., adverse reactions, abnormal organ function) reported	Level 2b (Oxford 2011 Evidence Grading)	Selection bias (non‐random allocation + no allocation concealment), performance bias (no blinding), measurement bias (unclear B‐ultrasound grading standards + unspecified detection instrument), follow‐up bias (no long‐term follow‐up beyond 4‐month treatment period), reporting bias (unreported safety outcomes)	(Zhu et al. 2018)
120 cases (57 in Observation Group/58 in Control Group; 5 cases lost to follow‐up: 3 in Observation Group, 2 in Control Group)	Not reported	Random sequence generation: SPSS‐generated random numbers + random number table method (patients numbered by admission time; random number seed and basis for allocation ratio not specified)	Not adopted	Core indicators: clinical therapeutic efficacy; liver function (ALT, AST, γ‐GT); lipid metabolism indicators (TC, TG, HDL‐C, LDL‐C); serum levels of Nesfatin‐1 and FGF21. Judgment criteria (referenced “TCM Diagnosis and Treatment Expert Consensus on Nonalcoholic Fatty Liver Disease”): Cure: liver morphology and parenchyma normalized by abdominal CT. Marked effect: severe fatty liver improved to mild by abdominal CT. Effective: severe → moderate or moderate → mild fatty liver by abdominal CT. Ineffective: no improvement in fatty liver severity. Total effective rate = (cure + marked effect + effective)/total cases × 100%. Measurement method: Liver function and lipid indicators: fasting venous blood (5 ml) detected by automatic biochemical analyzer. Nesfatin‐1/FGF21: fasting venous blood (3 mL) centrifuged at 3000 r/min for 30 min; measured by enzyme‐linked immunosorbent assay (ELISA); kits from Shanghai Jingke Reagent Co. Ltd. (Nesfatin‐1) and Chemicon (USA) (FGF21). Measurement time points: before treatment and after 2 courses of treatment (120 days total; 60 days per course)	Prespecified indicators: not reported (no adverse reaction monitoring or safety indicator assessment mentioned). Collection method: not reported. Follow‐up duration: 120 days (2 courses of treatment); 5 cases lost to follow‐up due to poor compliance. Results: no safety‐related data (e.g., adverse reactions, abnormal organ function) reported	Level 2b (Oxford 2011 Evidence Grading)	Selection bias (no allocation concealment), performance bias (no blinding), follow‐up bias (no long‐term follow‐up beyond 120‐day treatment period), reporting bias (unreported safety outcomes), attrition bias (5 cases lost to follow‐up)	(He et al. 2022)
71 cases (36 in Observation Group/35 in Control Group)	Not reported	Random sequence generation: Random number table method (explicitly stated; no information on independent sequence generation)	Not adopted	Core indicators: clinical therapeutic efficacy; liver function (ALT, AST); blood lipid indicators (TG, TC, LDL‐C, HDL‐C). Judgment criteria: Marked effect: liver echo and size basically normal by B‐ultrasound; liver density normalized by CT; clinical symptoms and signs disappeared. Effective: distal attenuation not significant, near‐field echo enhanced, and intrahepatic tubular structures visible by B‐ultrasound; liver density improved by CT; clinical symptoms and signs relieved. Ineffective: failed to meet the above criteria. Total effective rate = (marked effect + effective)/total cases × 100%. Measurement method: ALT/AST: detected by enzyme method. TG/TC/LDL‐C/HDL‐C: Measured by Hitachi 7600 automatic biochemical analyzer (Japan) with matching kits. Measurement time points: before treatment and after 2 months of continuous treatment	Prespecified indicators: not reported (no adverse reaction monitoring mentioned explicitly). Collection method: not reported. Follow‐up duration: 2 months (continuous treatment period). Results: no safety‐related data (e.g., adverse reactions, abnormal organ function) reported	Level 2b (Oxford 2011 Evidence Grading)	Selection bias (no allocation concealment), performance bias (no blinding), measurement bias (unclear B‐ultrasound/CT grading standards for efficacy evaluation), follow‐up bias (no long‐term follow‐up beyond 2‐month treatment period), reporting bias (unreported safety outcomes)	(Su 2021)
90 cases (45 in Treatment Group/45 in Control Group)	Not reported	Random sequence generation: Random grouping (specific method not specified)	Not adopted	Core indicators: liver function (ALT, AST, γ‐GT); clinical therapeutic efficacy. Judgment criteria (referenced “Guidelines for the Diagnosis and Treatment of Alcoholic Liver Disease [2010 Revised Edition]”): Cure: clinical symptoms and signs disappeared; syndrome score reduced by > 95%; liver B‐ultrasound image normal; liver function indicators normalized. Effective: clinical symptoms and signs significantly improved; syndrome score reduced by 30%–95%; at least 2 B‐ultrasound indicators reduced by ≥ 1 point compared with baseline; liver function basically normal. Ineffective: symptoms and signs unchanged, aggravated, or failed to meet the above criteria. Total effective rate = (cure + effective)/total cases × 100%. Measurement method: liver function indicators (ALT, AST, γ‐GT) detected by laboratory tests; liver morphology evaluated by B‐ultrasound. Measurement time points: before treatment and after 8 weeks of continuous treatment (2 courses, 4 weeks per course)	Prespecified indicators: adverse reactions during treatment (e.g., gastrointestinal reactions, abnormal liver/renal function). Collection method: active monitoring and recording of adverse reactions during treatment. Follow‐up duration: 8 weeks (2 courses of treatment). Results: no adverse reactions occurred in either group during treatment	Level 2b (Oxford 2011 Evidence Grading)	Selection bias (unclear randomization method + no allocation concealment), performance bias (no blinding), measurement bias (unclear B‐ultrasound scoring standards for efficacy evaluation), follow‐up bias (no long‐term follow‐up beyond 8‐week treatment period)	(Xuan 2019)
80 cases (37 in Treatment Group/35 in Control Group; 8 cases lost to follow‐up: 3 in Treatment Group, 5 in Control Group)	Not reported	Random sequence generation: SPSS‐generated random numbers +1:1 allocation (random number generation by SPSS 22.0 software; no specification of random number seed or allocation ratio basis)	Single‐blind (patient‐blinded). Implementation details: PLACEBO (composed of flour, excipients, and spices) was consistent with Chaihu Shugansan granules in color, texture, and taste; outcome assessors not blinded; no mention of blinding maintenance until study end	Core indicators: clinical efficacy of NAFLD; BMI; liver function (ALT, AST, γ‐GT); blood lipid indicators (TC, TG, HDL‐C, LDL‐C); inflammatory factors (IL‐6, TNF‐α, IL‐1β, TLR‐4); intestinal flora (*Bifidobacterium*, *Lactobacillus*, *Enterobacter*, *Enterococcus*); liver Controlled Attenuation Parameter (CAP) and Liver Stiffness Measurement (LSM). Judgment criteria: Clinical efficacy (abdominal ultrasound): cure (disappearance of fatty liver features); marked effect (reduction of fatty liver grade by 2 levels: severe → mild); effective (reduction of fatty liver grade by 1 level: severe → moderate or moderate → mild); ineffective (failure to meet effective criteria). Clinical efficacy (CAP): cure (CAP ≤ 230 dB/m); marked effect (reduction of CAP grade by 2 levels); effective (reduction of CAP grade by 1 level); Ineffective (failure to meet effective criteria). CAP grading: Mild (230 < CAP ≤ 252 dB/m), Moderate (252 < CAP ≤ 283 dB/m), Severe (CAP > 283 dB/m). Total effective rate = (cure + marked effect + effective)/total cases × 100%. 3 Measurement method: Liver function and blood lipids: detected by Olympus AU400 automatic biochemical analyzer (Japan). Inflammatory factors (IL‐6, TNF‐α, IL‐1β): Measured by ELISA (Invitrogen, USA). TLR‐4: detected by flow cytometry (FACS Calibur, BD, USA). Intestinal flora: 16S rRNA V3–V4 region amplification and sequencing (Guangzhou Kingmed Diagnostics). Abdominal ultrasound: Siemens Juniper Ultrasound System. CAP and LSM: FibroScan 502 transient elastography system (Echosens, France). 4 Measurement time points: before treatment and after 12 weeks of continuous treatment	Prespecified indicators: adverse reactions (e.g., gastrointestinal reactions, dry mouth, anorexia, diarrhea). Collection method: observed in accordance with Good Clinical Practice (GCP) requirements; regular follow‐up (1–2 times/week) during treatment. Follow‐up duration: 12 weeks (continuous treatment period). Results: 2 cases of gastrointestinal reactions (mild abdominal distension, diarrhea) in the Treatment Group (incidence 5.4%); 2 cases of dry mouth/anorexia/diarrhea in the Control Group (incidence 5.7%); no serious adverse events; no statistically significant difference in adverse reaction rates between the two groups (*p* > 0.05)	Level 2b (Oxford 2011 Evidence Grading)	Selection bias (no allocation concealment), performance bias (outcome assessors not blinded), attrition bias (8 cases lost to follow‐up), follow‐up bias (no long‐term follow‐up beyond 12‐week treatment period)	(Xie et al. 2021)
None (in silico study)	Not applicable (no human samples in in silico studies)	Not applicable (no human intervention)	Not applicable (no human intervention)	Core indicators: active components of Chaihu Shugan Powder; common targets of the formula for T2DM, NAFLD, and DD; enriched signaling pathways; molecular docking affinity between active components and key targets. Judgment criteria: g Active components: oral bioavailability (OB) ≥ 30% and drug‐likeness (DL) ≥ 0.18 (conventional thresholds for network pharmacology). h Key targets: top 5 by degree value (IL6, TNF, IL1B, AKT1, VEGFA). i Signaling pathways: KEGG enrichment analysis with *p* ≤ 0.01. j Molecular docking: Binding energy ≤ −6.0 kcal/mol (indicating stable binding). 3 Measurement method: Active components/targets: screened via TCMSP, GeneCards, and UniProt databases; network constructed by Cytoscape 3.7.0. Pathway enrichment: DAVID database (GO/KEGG analysis). Molecular docking: Autodock vina 1.1.2 and Pymol 2.4.0 software (preprocessing of 2D/3D structures, docking validation). 4 Measurement time point: not applicable (in silico simulation only)	Prespecified indicators: not applicable (no human medication or intervention). Results: no safety data available (no in vivo/in vitro toxicity evaluation)	Level 4 (Oxford 2011 Evidence Grading)	Data source bias (partial software parameters not fully specified), network construction bias (screening thresholds lack personalized justification), docking bias (molecular docking parameters not detailed), extrapolation bias (human applicability not verified by clinical/in vitro experiments)	(Chang et al. 2023)

Single‐component compounds, limited by pharmacokinetic properties and toxicity issues, still require optimization of translational pathways through basic research and more clinical randomized controlled trials for support. As pentacyclic triterpenoid oleanane‐type derivatives, SSs exhibit poor oral absorption and low bioavailability, while injection administration causes significant hemolytic toxicity. However, published studies have shown that drugs with poor intestinal absorption can achieve therapeutic effects by regulating gut microbiota balance via the gut‐microbiota‐liver axis, or be converted into metabolites that exert biological activity under the action of gastric acid and gut microbiota (Li et al. 2018; Song et al. 2019; Zhou et al. 2021). Previous research has also demonstrated that with the marked improvement in LC–MS/MS detection sensitivity, SSA, SSD, and SSC in rat plasma are rapidly absorbed into the bloodstream within 30 min after oral administration of *Bupleurum* extracts (Xu et al. 2012).

## 4 Cross‐Organ Protective Effect

### 4.1 Anti‐Tumor Effect

If MASLD is not promptly intervened, it may progress to hepatocellular carcinoma (HCC) and has been confirmed by multiple statistical analyses to be significantly associated with colorectal cancer, breast cancer, and gastric cancer. Severe MASLD independently increases colorectal tumor risk, with a predilection for left colon tumors (Reja et al. 2020; Zeng et al. 2022; Zheng et al. 2018). SSD exhibits promising anti‐tumor activity with translational potential for MASLD‐related malignancies. Evidence from in vitro cell experiments shows that SSD can specifically enhance the radiosensitivity of hepatoma cells, with no significant effect on the radiosensitivity of normal hepatocytes (*p* > 0.05), suggesting that SSD may not only improve the efficacy of radiotherapy for liver cancer but also reduce radiation‐induced damage to normal liver tissue. This effect may be achieved by inhibiting the phosphorylation of the AMPK‐mTOR pathway, promoting autophagy in hepatoma cells, and thereby suppressing the proliferation of hepatoma cells (Tian et al. 2019; Wang et al. 2021). The activation of the SHH pathway and GLI protein family, as well as the SUMOylation of general proteins, all play important roles in tumorigenicity and cancer. Preclinical in vitro and in vivo experiments have demonstrated that under hypoxic conditions, SSD can block the SUMOylation of GLI proteins by specifically upregulating the expression of SENP5, thereby inhibiting the growth of hepatoma cells and enhancing their sensitivity to chemotherapy (Jiang 2022; Zhang et al. 2019). Similarly, cell experiments have found that SSD can also inhibit the proliferation of hepatoma cells and induce their apoptosis by blocking the phosphorylation of STAT3 and suppressing the expression of downstream transcription factors C/EBPβ and COX‐2 (Ren et al. 2019). Animal experiments have further shown that SSD can alleviate liver damage in SD rats with diethylnitrosamine (DEN)‐induced carcinogenesis; compared with the DMSO control group, SSD downregulates the expression of STAT3 and COX‐2 in rat liver cancer tissues and inhibits the formation of liver cancer in rats (Yaxin et al. 2011). For colorectal cancer, Lee et al. confirmed through in vitro and in vivo experiments that SSD can induce autophagy and apoptosis of colorectal cancer cells via the p38/ERK–MAPK signaling pathway, effectively inhibiting the proliferation of cells and their lung metastasis (Lee, Mun, et al. 2024). For breast cancer, SSD can induce breast cancer cell apoptosis by blocking the fusion of autophagosomes with lysosomes, activating the MAPK pathway to significantly upregulate p‐p38 levels, and promoting the cleavage of caspase‐3/PARP; it can also reverse doxorubicin and paclitaxel resistance by downregulating MDR1/P‐gp or blocking the Hippo/YAP pathway, and inhibit cancer cell proliferation by suppressing the JAK2/STAT3 pathway and downregulating key glycolytic enzymes such as GLUT1 and LDHA (Fu et al. 2020; Li, Zhang, and Li 2017; Wang and Shi 2024; Zhu et al. 2024). Regarding gastric cancer, SSD can enhance the sensitivity of gastric cancer cells to the anticancer drug cisplatin by promoting the formation of apoptosis‐related proteins and autophagosomes and inhibiting the NF‐κB signaling pathway (Hu et al. 2021). A study by Yang et al. found that the combination of saikosaponins and programmed death receptor 1/programmed death ligand 1 (PD‐1/PD‐L1) inhibitors can inhibit the proliferation, invasion, and migration of gastric cancer cells, downregulate Bcl‐2 and PI3K/AKT pathway‐related proteins, and promote the apoptosis of gastric cancer cells (Yang et al. 2025). Additionally, SSD can inhibit the malignant phenotype of gastric cancer by suppressing glycolysis and the expression of YAP1 and c‐Myc (Zhu et al. 2024).

All the aforementioned effects of SSD on tumors are based on preclinical studies. Among these, the mechanisms by which “SSD enhances chemo/radiosensitivity and reverses drug resistance” and “improves gastric cancer efficacy when combined with PD‐1/PD‐L1 inhibitors” hold potential for near‐term clinical application and may provide an auxiliary treatment direction for MASLD patients with concurrent tumors. However, these are currently hypothetical perspectives; only after verifying their safety and efficacy through human clinical trials can they be further incorporated into MASLD clinical management strategies.

### 4.2 Cardiovascular System Protection

Patients with MASLD have a significantly increased risk of developing cardiovascular and cerebrovascular events such as coronary heart disease (CHD), myocardial infarction (MI), and stroke. The shared pathophysiological mechanisms between MASLD and CVD include IR, dyslipidemia, chronic low‐grade inflammation (elevated IL‐6 and TNF‐α), oxidative stress, gut microbiota dysbiosis, and genetic factors. These factors not only accelerate the progression of atherosclerosis but also lead to a positive correlation between the severity of MASLD and the degree of coronary artery lesions (Arslan and Yenerçağ 2020; Wang, Wang, et al. 2023; Zisis et al. 2025).

Saikosaponins show preclinical cardioprotective potential relevant to MASLD management: SSA inhibits oxidized low‐density lipoprotein‐induced foam cell formation and NLRP3 inflammasome activation via PI3K/AKT/NF‐κB pathway suppression (D. He et al. 2016); SSD alleviates doxorubicin‐induced cardiotoxicity through p38‐MAPK inhibition (Zhang et al. 2022). Both saponins exert anti‐atherosclerotic effects via TLR4/NF‐κB modulation and cholesterol metabolism regulation (Zhang et al. 2022).

All the above findings are primarily based on preclinical studies: the anti‐CVD effects of SSA/SSD have only been verified in cell or animal models, and no human clinical trials have been conducted in MASLD patients with comorbid CVD. From a clinical management perspective, this undeniable link mandates a paradigm where cardiovascular risk assessment is non‐negotiable in MASLD care. The translational potential of SSs lies in their adjunctive role, and this remains a future possibility pending safety and efficacy data from human trials. For now, the cornerstone of management is to treat the MASLD patient as a cardiovascular patient first. This suggests that SSs may treat patients with MASLD and comorbid cardiovascular disease by inhibiting foam cell formation and reducing vascular inflammation via SSs.

### 4.3 Renal Protection

MASLD is significantly associated with chronic kidney disease (CKD), and the prevalence of CKD in MASLD patients is approximately twice that in the general population. The core mechanisms lie in IR, metabolic disorders, inflammation, oxidative stress, and endothelial dysfunction—all of which may increase the risk of CKD by impairing renal filtration function and hemodynamics (Byrne and Targher 2015; Umbro et al. 2021). Preclinical studies confirm renal protective effects of saikosaponins: SSA/SSD activate PI3K/AKT/Nrf2 to reduce oxidative stress and inflammation in CKD mice (Huang et al. 2023). SSD upregulates SIRT3 to enhance antioxidant capacity and inhibit MAPK/NF‐κB signaling, protecting tubular epithelial cells from high‐glucose damage (Ma et al. 2015; Zhao et al. 2015). These findings suggest that SSD may exert renal protective effects by improving oxidative stress and inhibiting inflammation.

Translational relevance to MASLD management is limited by the lack of human trials in comorbid patients. Current guidelines, however, provide clear actionable steps: annual monitoring of estimated glomerular filtration rate (eGFR) and urinary albumin‐to‐creatinine ratio (UACR) for early CKD detection. For confirmed CKD, angiotensin receptor blockers, SGLT2 inhibitors, and PPAR‐γ agonists are recommended—agents proven effective for both CKD and MASLD (Bilson et al. 2024; Schnell et al. 2024). Saikosaponins' preclinical efficacy supports future trials, but they cannot yet be integrated into clinical practice.

### 4.4 Alleviation of Obesity‐Related Symptoms

Obesity and MASLD are epidemiologically linked, with MASLD prevalence exceeding 90% in severe obesity versus 25% in the general population (Younossi et al. 2019). Saikosaponins target this comorbidity in preclinical models: SSD reduces weight gain, hepatic steatosis, and dyslipidemia in obese mice by remodeling gut microbiota and activating PKA/AMPK/p38‐mediated lipolysis (Wang, Chen, et al. 2025). SSA improves insulin resistance via FGF21‐mediated metabolic regulation and autophagy activation (Lee, Noh, and Lee 2024).

Clinically, these findings await human validation. Current MASLD management prioritizes lifestyle intervention for weight control. For non‐responders, GLP‐1 receptor agonists are recommended—they reduce weight, improve hepatic steatosis, and lower cardiovascular risk, providing a proven strategy for obesity‐related MASLD (Handelsman et al. 2024).

### 4.5 Improvement of Type 2 Diabetes Mellitus

Type 2 diabetes mellitus (T2DM) affects over 60% of MASLD patients and accelerates progression to advanced fibrosis (Younossi et al. 2018). Saikosaponins address this comorbidity via multiple pathways: SSA enhances PI3K/Akt signaling to improve insulin sensitivity and reduce lipid accumulation (Lin et al. 2024). High‐dose SSD exhibits comparable efficacy to metformin by inhibiting FoxO1/PGC‐1α and AQP1/RhoA/ROCK pathways, reducing blood glucose and inflammation (Song et al. 2023; Xiang et al. 2023).

These findings are primarily based on preclinical studies: the ameliorative effects of SSA and SSD on T2DM‐related MASLD have only been verified in cell or animal models. No human clinical trials of SSs in patients with T2DM‐complicated MASLD have been conducted. They cannot be directly incorporated into the clinical treatment protocols for T2DM‐related MASLD at present, but based on existing experimental evidence, SSs show potential for treating patients with MASLD comorbid with T2DM. No clinical trials exist in T2DM‐MASLD patients, but guidelines recommend GLP‐1 receptor agonists (e.g., semaglutide), SGLT2 inhibitors (e.g., dapagliflozin), and pioglitazone—agents that target both T2DM and MASLD progression (Kernan et al. 2016; McGuire et al. 2021; Sattar et al. 2021).

### 4.6 Improvement of Hyperlipidemia

Hyperlipidemia and MASLD form a reciprocal pathogenic cycle: hepatic lipid deposition exacerbates oxidative stress and IR, further disrupting lipid metabolism (Cheng et al. 2024). SSs normalize lipid metabolism via LKB1‐AMPK activation (reducing TC/TG/LDL‐C), HMGCR inhibition, and mitochondrial fatty acid oxidation enhancement (Du et al. 2022; Zheng et al. 2023). Additionally, bioinformatic analyses confirm modulation of JAK–STAT and PI3K‐Akt pathways (Cheng et al. 2023).

From a clinical perspective, based on preclinical studies: the ameliorative effects of SSs on hyperlipidemia‐related MASLD have only been verified in RNA sequencing, metabolomic analysis, and cell experiments, and no human clinical trials of SSs in patients with MASLD complicated by hyperlipidemia have been conducted. Clinical management follows established guidelines: lifestyle intervention (low‐fat diet, omega‐3 supplementation) first, then maximum‐tolerated statins (e.g., atorvastatin) to reduce LDL‐C by 30%–50% (Handelsman et al. 2024).

### 4.7 Improvement of Immune System

Immune dysregulation drives MASLD progression from steatosis to fibrosis and HCC, mediated by innate (TLRs, NLRP3 inflammasome) and adaptive (CD8^+^T cell exhaustion, Th17 enrichment) immunity (Tilg et al. 2021; Yahoo et al. 2023). Experimental evidence shows that SSA inhibits T cell proliferation and arrests cell cycle at G0/G1 phase in Con A‐stimulated mice (Sun et al. 2009). Additionally, SSD upregulates CTLA‐4/IL‐10 via JAK–STAT/NF‐κB pathways to alleviate autoimmune hepatitis (Hao et al. 2020). These findings suggest that both SSA and SSD can alleviate MASLD by inhibiting the abnormal activation of the immune system.

From the aforementioned animal experiments, it has been shown that SSs may regulate the abnormal activation of the immune system, inhibit the proliferation and activation of T cells, and reduce cytotoxicity and liver inflammation. Gut microbiota modulation and bile acid receptor agonists for immune regulation require further trials. Saikosaponins' gut microbiota‐immune crosstalk effects offer an additional investigational avenue for MASLD management.

### 4.8 Central Nervous System Protection

Emerging evidence links MASLD to central nervous system (CNS) disorders (e.g., dementia, Parkinson's disease [PD]) via the gut microbiota‐gut‐brain axis, with shared inflammatory and oxidative stress pathways (Estrada et al. 2019). Research indicates that SSs can alleviate inflammation and oxidative stress, improving both MASLD and CNS‐related conditions (Ding et al. 2020). Patients with dyslipidemia often have an increased risk of Alzheimer's disease (AD), mild cognitive impairment (MCI), and dementia; additionally, a correlation between neurodegeneration and cognitive deficits has been observed in patients with metabolic syndrome‐related diseases (de la Monte et al. 2009). This association is relevant to holistic MASLD management, as CNS comorbidities impact quality of life.

Preclinical studies show saikosaponin‐mediated neuroprotection: SSA protects dopaminergic neurons in PD mice via TLR4/MyD88/NF‐κB inhibition (Liu et al. 2023). SSD alleviates hippocampal neuroinflammation and depression‐like behaviors by targeting HMGB1/TLR4/NF‐κB (Su et al. 2020). SSA modulates gut microbiota and monoamine neurotransmitters to improve depression (Wang, Li, et al. 2025).

Clinical translation is premature. Current research is limited to observational studies using tools like the Montreal Cognitive Assessment (Colognesi et al. 2020). While saikosaponins show promise for dual MASLD‐CNS protection, dedicated trials in comorbid patients are needed before clinical application.

## 5 Conclusion and Future Prospects

MASLD characterized as a hepatic manifestation of multi‐system metabolic disorders is driven by complex pathogenic mechanisms involving lipid metabolic imbalance, insulin resistance, inflammation and oxidative stress, gut microbiota dysbiosis, and extrahepatic organ crosstalk. SSs, the core bioactive constituents of *Bupleurum*species, have emerged as promising therapeutic agents for MASLD due to their multi‐target, multi‐pathway mechanisms. The present studies collectively highlight that SSs—particularly SSA and SSD—exert their protective effects through coordinated regulation of metabolic, inflammatory, and inter‐organ networks, with emerging evidence expanding their therapeutic scope beyond the liver.

To fully realize SSs' therapeutic potential, future research must address these challenges holistically. First, developing novel delivery systems—such as nano‐formulations, lipid‐based carriers, or prodrugs—could enhance bioavailability and reduce off‐target effects, bridging the gap between preclinical efficacy and clinical applicability. Second, elucidating the molecular mechanisms underlying SSs' “gut‐liver‐brain” axis regulation and their interactions with extrahepatic organs (e.g., adipose tissue, skeletal muscle) in metabolic syndrome will clarify their multi‐target actions and identify synergistic combinations with existing therapies (e.g., GLP‐1 receptor agonists, SGLT2 inhibitors). Finally, large‐scale, multi‐center clinical trials are needed to validate optimal dosing, long‐term safety, and real‐world effectiveness across diverse patient populations. In‐depth analysis of the molecular regulatory mechanisms by which SSs exert effects through cross‐organ networks such as the “gut‐liver‐brain axis,” as well as their synergistic therapeutic roles in other extrahepatic organs (e.g., cardiovascular, kidney, and metabolic syndrome) and complicated comorbidities, remains necessary.

In summary, SSs demonstrate significant advantages in preventing and treating MASLD‐related diseases, leveraging their multi‐targeted intervention and cross‐organ protection properties. However, numerous challenges persist. It is anticipated that through continued in‐depth research, SSs will attain greater therapeutic advantages in the management of MASLD.

## Author Contributions

J.X. contributed to the conception and design of this work, and wrote and revised the manuscript. Z.J. provided guidance on the organization and content of the manuscript. X.L. helped in preparation of the figures. Y.C. and W.X. helped to revise the structure and language of the manuscript. W.T. provided guidance, edited and revised the manuscript, and is responsible for all aspects of the manuscript. All authors contributed to the manuscript and approved the submitted version.

## Funding

This study was supported by the Intra‐institutional Scientific Research Fund of Liyuan Hospital, Tongji Medical College, Huazhong University of Science and Technology (by 2023LYYYCXTD0002).

## Conflicts of Interest

The authors declare no conflicts of interest.

## Data Availability

This article is a review paper and does not report any new primary data. All data analyzed or discussed in this review were derived from previously published studies and publicly available databases, which are cited throughout the text. No new datasets were generated or analyzed during the current study.
